# Melatonin prevents lung injury by regulating apelin 13 to improve mitochondrial dysfunction

**DOI:** 10.1038/s12276-019-0273-8

**Published:** 2019-07-04

**Authors:** Lu Zhang, Fang Li, Xiaomin Su, Yue Li, Yining Wang, Ruonan Fang, Yingying Guo, Tongzhu Jin, Huitong Shan, Xiaoguang Zhao, Rui Yang, Hongli Shan, Haihai Liang

**Affiliations:** 1 Department of Pharmacology (State-Province Key Laboratories of Biomedicine-Pharmaceutics of China, Key Laboratory of Cardiovascular Research, Ministry of Education), College of Pharmacy, Harbin Medical University, Harbin, 150081 Heilongjiang PR China; 2 Northern Translational Medicine Research and Cooperation Center, Heilongjiang Academy of Medical Sciences, Harbin Medical University, Harbin, 150081 Heilongjiang PR China; 3 The 962nd Hospital of Nephrology Department, Harbin, 150081 Heilongjiang PR China; 4 Department of Physiology, Shenyang Medical College, Shenyang, 110034 Liaoning PR China

**Keywords:** Diseases, Therapeutics

## Abstract

Pulmonary fibrosis is a progressive disease characterized by epithelial cell damage, fibroblast proliferation, excessive extracellular matrix (ECM) deposition, and lung tissue scarring. Melatonin, a hormone produced by the pineal gland, plays an important role in multiple physiological and pathological responses in organisms. However, the function of melatonin in the development of bleomycin-induced pulmonary injury is poorly understood. In the present study, we found that melatonin significantly decreased mortality and restored the function of the alveolar epithelium in bleomycin-treated mice. However, pulmonary function mainly depends on type II alveolar epithelial cells (AECIIs) and is linked to mitochondrial integrity. We also found that melatonin reduced the production of reactive oxygen species (ROS) and prevented apoptosis and senescence in AECIIs. Luzindole, a nonselective melatonin receptor antagonist, blocked the protective action of melatonin. Interestingly, we found that the expression of apelin 13 was significantly downregulated in vitro and in vivo and that this downregulation was reversed by melatonin. Furthermore, ML221, an apelin inhibitor, disrupted the beneficial effects of melatonin on alveolar epithelial cells. Taken together, these results suggest that melatonin alleviates lung injury through regulating apelin 13 to improve mitochondrial dysfunction in the process of bleomycin-induced pulmonary injury.

## Lung disease: managing misbehaving mitochondria

The hormone melatonin could protect lung cells from the damage associated with respiratory diseases such as pulmonary fibrosis. Several studies have linked such damage with abnormal activity of the mitochondria, with these essential metabolic organelles churning out damaging ‘reactive oxygen species’ (ROS), compounds that induce premature cell aging and death. Melatonin can mitigate ROS production, and researchers led by Haihai Liang at China’s Harbin Medical University have demonstrated that it can prevent injury to airway epithelial cells in a mouse model of lung disease. Melatonin treatment countered much of the damage, resulting in significantly longer survival, and the team identified a target molecule in the mitochondria that may be responsible for this effect. This approach could offer hope for a family of diseases with a poor prognosis and limited treatment options.

## Introduction

Direct or indirect stimulating factors lead to the diffuse alveolar damage, which increased alveolar capillary membrane permeability, excessive pulmonary inflammation, and alveolar epithelial cell apoptosis termed lung injury. In addition, excessive apoptosis of type II alveolar epithelial cells (AECIIs) impairs the epithelial barrier^1^, accompanied by the development of senescence and inflammation in alveolar epithelial cells^2^. Thus, it is urgent to explore therapeutic methods targeting the repair of alveolar epithelial cells during lung injury.

Mitochondrial dysfunction and reactive oxygen species (ROS) overproduction have been proposed to mediate the pathogenesis of many diseases, including lung injury^3,4^. In a previous study, Chen et al.^5^ showed that intravenous administration of exogenous irisin protected against IR-induced lung injury via the improvement of mitochondrial function. In addition, suplatast tosilate protected against hyperoxic lung injury by decreasing the degree of oxidative stress induced by ROS, particularly through its ability to scavenge hydroxyl radicals^6^. Thus far, drug therapies used for the clinical treatment of lung injury usually lack effectiveness. Therefore, it is urgent to explore new effective methods for regulating mitochondrial function to ameliorate lung injury.

Melatonin (N-acetyl-5-methoxytryptamine, MLN) is a neuroendocrine hormone that is synthesized in the pineal gland and other organs^7^. Accumulating evidence has shown that MLN is involved in a variety of diseases. MLN offers protection against ROS as individual chemical entities through a wide variety of mechanisms, including electron transfer, hydrogen transfer, radical adduct formation, metal chelation, and the repair of biological targets. Experimental evidence obtained from both human and rodent studies has demonstrated that MLN improves pulmonary function, for example, regulating PARP1 to control the senescence-associated secretory phenotype (SASP) in human fetal lung fibroblast cells^8^ and attenuating pulmonary hypertension in chronically hypoxic rats^9^. A recent study showed that MLN alleviated intracerebral hemorrhage-induced secondary brain injury in rats via suppressing apoptosis, inflammation, oxidative stress, DNA damage, and mitochondrial injury^10^. Although MLN could protect against lung diseases, the exact role of MLN in lung injury, as well as the underlying mechanisms, remains ambiguous. Other reports showed that MLN prevented abnormal mitochondrial dynamics resulting from cadmium neurotoxicity^11^. Thus, we speculated that MLN protected against lung injury through regulating mitochondria.

The G protein-coupled receptor APJ and its homologous ligand, apelin, are widely expressed in different tissues and participate in different physiological processes, such as angiogenesis, cardiovascular function, fluid homeostasis, and energy metabolism regulation^12,13^. Recent studies have shown a prominent role of apelin in lung diseases^14,15^. Neonatal rats exposed to prolonged hypoxia with apelin attenuated lung inflammation and RVH, increased cGMP levels and alveolarization, and promoted pulmonary angiogenesis^16^. In addition, it has been reported that apelin could modulate mitochondrial function by inhibiting dynamin-related protein 1^17^ and mitochondrial ROS^18^ in myocardial ischemia-reperfusion injury. However, whether apelin is involved in the mechanism by which MLN prevents lung injury remains to be explored.

Therefore, we focused on exploring whether MLN treatment can prevent pulmonary injury as well as investigating the underlying molecular mechanisms. The aim of this study was to identify the beneficial role of MLN in reducing pulmonary dysfunction and to reveal a new approach for pulmonary disease treatment in the clinic.

## Materials and methods

### Chemicals and reagents

Bleomycin (BLM) was purchased from Selleck (Shanghai, China). MLN, luzindole, apelin 13, and ML221 were purchased from Sigma Chemical Co. (St. Louis, MO).

### Animals

C57BL/6 male mice (8–10 weeks; 20–25 g) were used in the present study. All in vivo animal procedures were conducted with the approval and guidance of the University Committee on the Use and Care of Animals. Experimental mice were provided standard rodent chow and water and were housed in a controlled environment near a window for access to normal sunlight. Mice were divided randomly into four groups: PBS, BLM, bleomycin + melatonin, and bleomycin + melatonin + ML221. To establish the pulmonary injury model, experimental mice were administered a single dose of 5 mg/kg body weight BLM or PBS by intratracheal instillation; after 24 h, BLM + MLN group mice received intraperitoneal injections of MLN (5 mg/kg body weight per day for 3 weeks). BLM + MLN + ML221 group mice received intraperitoneal injections of ML221 (150 μg/kg body weight per day for 3 weeks). The dose of MLN used in the present study was in accordance with that used in a previously published study^19^. We intraperitoneally injected MLN at noon, when endogenous MLN secretion was the lowest. Finally, mice from each group were sacrificed on day 21, and samples were collected.

### Cell culture

TC-1 cells were purchased from Shanghai Enzyme Research Biotechnology Co., Ltd (Shanghai, China). TC-1 cells were cultured in 25 cm^2^ cell culture flasks, 6-well plates, 12-well plates, or 24-well plates with RPMI 1640 (Biological Industries) supplemented with 10% fetal bovine serum (FBS, Biological Industries) and 1% penicillin/streptomycin (Beyotime, Shang Hai, China). The cells were maintained at 37 °C with 5% CO_2_ and 95% air. After starvation in serum-free medium for 12 h, cells were separately administered MLN (10 nM, 1 μM, and 100 μM), luzindole (10 μM), apelin 13 (100 nM), or ML221 (10 μM) for 24 h and treated with H_2_O_2_ (200 μM) for 12 h.

### Protein isolation and western blotting

Total protein lysates from TC-1 cells or lung tissue were prepared in RIPA lysis buffer (50 mM Tris-HCl, pH 7.4; 150 mM NaCl; 1 mM EDTA; 1% Triton X-100; 1% sodium deoxycholate; 0.1% sodium dodecyl sulfate; and 1 mM phenylmethylsulfonyl fluoride) supplemented with a protease inhibitor cocktail, incubated for 30 min on ice, and centrifuged for 15 min at 13,500 rpm. Protein concentrations were determined with the bicinchoninic acid (BCA) protein assay reagents. Protein samples were separated by SDS-PAGE and transferred to nitrocellulose filter membranes. Membranes were blocked using 1× PBS containing 0.1% Tween 20 and 5% w/v nonfat dry milk. The following primary antibodies were purchased from Proteintech (Wuhan, China): anti-E-cadherin (20874-1-AP), anti-p53 (1044-1-AP), anti-p21 CDKN1A (10355-1-AP), anti-Bcl2 (12789-1-AP), anti-BCL-2-associated X protein (Bax) (50599-2-Ig), anti-cytochrome C (Cyt-c) (10993-1-AP), and anti-β-actin (60008-1-Ig). After incubation with the corresponding anti-mouse/-rabbit secondary antibodies, immunoblots were developed using an Odyssey CLx imager (Gene Company Limited, Hongkong, China). Signal intensities were quantified with Image Studio software. β-actin was used as the loading control.

### Quantitative RT-PCR

RNA was isolated using TRIzol reagent according to the manufacturer’s instructions. With 5 × All-In-One, RNA was reverse transcribed to complementary DNA (cDNA). Diluted cDNA was then subjected to quantitative RT-PCR with gene-specific primers in the presence of Eva Green on a 7500 Fast real-time PCR system using β-actin as the control. The specific primers used in this study were as follows: IL1β (F: GAAATGCCACCTTTTGACAGTG; R: TGGATGCTCTCATCAGGACAG); IL6 (F: CACAGCAAGGCCTAGGAAAG; R: TTGGTTCAGCCACTGCCGTA); TNFα (F: TGGATGCTCTCATCAGGACAG; R: TGGATGCTCTCATCAGGACAG); Cdh1 (F: AGACTTTGGTGTGGGTCAGG; R: AGACTTTGGTGTGGGTCAGG); Sftpc (F: CAGCTCCAGGAACCTACTGC; R: CACAGCAAGGCCTAGGAAAG).

### TUNEL assay

Cells were fixed in 4% paraformaldehyde in PBS and permeabilized with 0.1% Triton X-100 in 0.1% sodium citrate. An in situ apoptotic cell death detection kit (fluorescein, Roche Applied Science) based on the TUNEL assay was used according to the manufacturer’s instructions to detect apoptotic cells. Negative controls were included in each case by omitting the TUNEL enzyme terminal deoxynucleotidyl transferase reaction mixture and incubating the cells with the labeling solution. PBS containing 5 μg/ml 4′,6-diamidino-2-phenylindole (DAPI; Vector Laboratories) was prepared to stain nuclei. The number of TUNEL-positive cells (green cells) and the total number of cells (blue cells) were counted.

### Evans blue staining

Mice were intraperitoneally injected with Evans blue solution (1 ml/kg of 3% Evans blue solution in PBS) for 30 min before euthanization. The amount of Evans blue was measured in whole lungs from mice in the PBS, BLM and BLM + MLN groups and normalized to the Evans blue concentration in methanamide for 24 h to generate an Evans blue index.

### Immunohistochemistry

Mouse lung tissues were fixed in 4% paraformaldehyde for 1 week and dehydrated for 24 h. Paraffin-embedded lung tissues were sliced into 4 μm sections. Immunohistochemical staining was performed using the antibody against E-cadherin. Quantification of the immunopositive area was determined by Image J analysis as the ratio of the positive-stained area to the total area.

### Measurement of intracellular ROS

TC-1 cells were incubated for 30 min with 10 µM 2′,7′-dichlorodihydrofluorescein diacetate (H2DCF-DA) probe, a fluorogenic dye used for measuring ROS levels within cells. After fixation in 4% paraformaldehyde and PBS washes, cells were incubated in DAPI for 10 min to stain nuclei. Fluorescence signals were analyzed by a fluorescence microscope.

### Senescence-associated β-galactosidase assay

Senescence-associated β-galactosidase activity was assessed by using a Senescence β-Galactosidase Staining Kit (Beyotime, Shanghai, China) according to the manufacturer’s protocol. Cells were washed with PBS and fixed with 4% paraformaldehyde for 15 min at room temperature. Then cells were washed three times with PBS. Next, cells were incubated overnight at 37 °C in the dark with working solution containing 0.05 mg/ml 5-bromo-4-chloro-3-indolyl β-D-galactopyranoside (X-gal). On the following day, cell staining was visualized and imaged using a microscope.

### ATP determination

The level of ATP was measured by an ATP assay kit (Beyotime, Shanghai, China) based on a bioluminescence technique. Cells were washed once with PBS and transferred to lysis buffer. The liquid supernatant was centrifuged at 12,000 × *g* for 5 min at 4 °C and mixed with ATP detection buffer before analysis by luminescence spectrometry. The final ATP content in each sample was normalized to its protein concentration measured by the BCA Protein Assay Kit.

### Transmission electron microscopy

Lung tissues were sliced into ultrathin sections and fixed with 4% paraformaldehyde and 1% glutaraldehyde in PBS at 4 °C overnight. The preparations were washed and dehydrated with increasing concentrations of ethanol, followed by embedding and sectioning. The tissue slices were examined under an H7650 transmission electron microscope (Hitachi, Japan). For each treatment, micrographs of at least 15 unique cells were acquired.

### Mitochondrial respiration measurements

Whole-cell respiratory function was determined by high-resolution respirometry (Oxygraph-2k; Oroboros Instruments, Innsbruck, Austria) with procedures described in a previous study. The protocol was designed with oligomycin-uncoupler-inhibitor titrations to evaluate the effects of uncouplers on the maximal uncoupled respiratory capacity of the electron transfer system. Briefly, titration of the uncouplers (1 mM uncouplers: FCCP, niclosamide, and BAM15) was performed in steps of 1 µl, corresponding to a stepwise increase in the final uncoupler concentration of 0.5 mM in intervals of 120 s until the maximum noncoupled flux was attained. Routine respiration (Routine) was recorded after stabilization. Maximal mitochondrial respiration in the mitochondria was calculated by subtracting the oxygen consumption rate (OCR) elicited by 2 μg/ml oligomycin from the maximum OCR induced by the mitochondrial uncouplers. The reserve respiratory capacity is equal to the difference between the maximum OCR and the routine OCR. Inhibitors of complexes CI (rotenone, 0.5 μM) and CIII (antimycin A, 2.5 μM) were administered to measure the residual oxygen consumption.

### Statistical analysis

All values are expressed as the means ± SEMs. Differences between two groups were determined via Student’s *t*-test. A two-tailed value of *P* < 0.05 was considered statistically significant. Data were analyzed using GraphPad Prism 6.0.

## Results

### Protective function of MLN against BLM-induced lung injury in mice

**Fig. 1:**
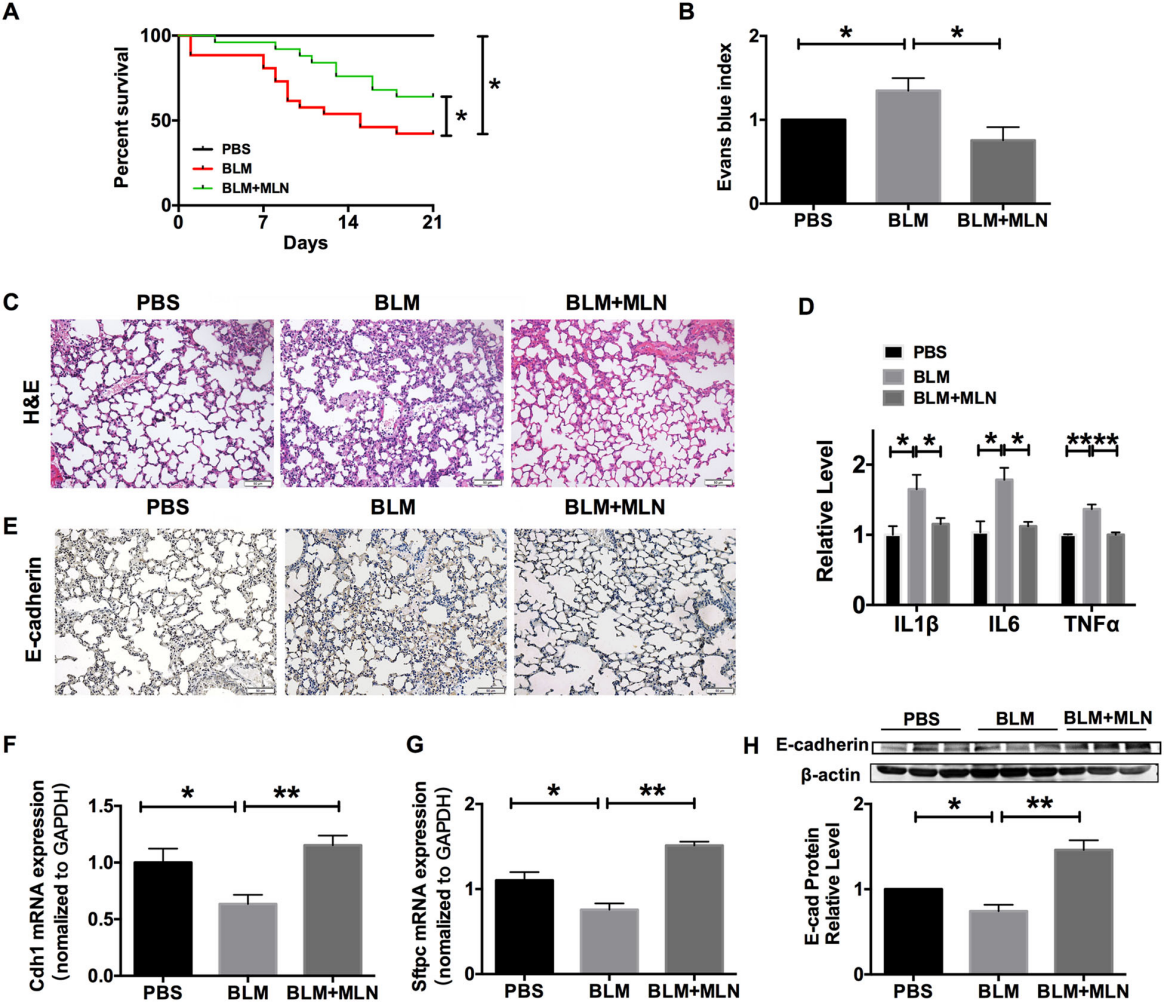
Melatonin increased survival and improved pulmonary epithelial function in response to bleomycin. Mice were intratracheally treated with PBS or bleomycin (BLM) with or without melatonin (MLN), (PBS group: *n* = 25, BLM group: *n* = 26, BLM + MLN group: *n* = 25). **a** Survival plots of mice in the PBS, BLM, and BLM + MLN groups. *P < 0.05. **b** Mice were intraperitoneally injected with Evans blue solution (1 ml/kg 3% Evans blue solution in PBS) for 30 min before euthanization. The amount of Evans blue was measured in the whole lungs of mice in the PBS, BLM, and BLM + MLN groups and normalized to the Evans blue concentration in methanamide to generate an Evans blue index. *n* = 3; *P < 0.05. **c** H&E staining of representative lung sections from mice in the PBS, BLM, and BLM + MLN groups. Scale bars, 50 μm. **d** The mRNA expression of inflammatory factors (IL1β, IL6, and TNFα). *n* = 4; *P < 0.05, **P < 0.01. **e** The Immunohistochemical assay of E-cadherin in lung samples from mice in the PBS, BLM, and BLM + MLN groups. Scale bar, 50 μm. **f–g** Quantitative RT-PCR was performed to analyze the mRNA expression of E-cadherin (Cdh1) and Sftpc. *n* = 5; *P < 0.05, **P < 0.01. **h** E-cadherin, a marker of alveolar epithelial cells, was identified by immunoblot analysis. *n* = 6; *P < 0.05, **P < 0.01

To determine the function of MLN in lung injury, C57BL/6 mice were administered bleomycin (BLM, 5 mg/kg) with or without MLN (MLN, 5 mg/kg/day for 3 weeks). In contrast to the BLM group, the group treated with MLN exhibited a substantial survival benefit, with a significant increase in survival from 42.3% in the BLM group to 64% in the MLN-treated group (Fig. 1a). This survival benefit suggested that MLN potentially ameliorated lung injury induced by BLM. As an additional measure of epithelial permeability, mice subjected to different treatments were intraperitoneally injected with Evans blue (1 ml/kg 3% Evans blue solution in PBS) for 30 min. Mice were then euthanized, whole lungs were collected, and the Evans blue concentration was measured. BLM group mice showed a significantly higher level of Evans blue than PBS group mice, and MLN significantly decreased the Evans blue index (Fig. 1b), indicating that MLN could recover epithelial barrier function after BLM treatment. BLM group mice exhibited significant irregularities in the alveolar structure, an increased number of inflammatory cells and accelerated expression of inflammatory factors including IL1β, IL6, and TNFα, in the lung; however,MLN reversed the significant abnormality in static lung compliance induced by BLM (Fig. 1c, d).

Histological sections from the lungs of mice in different groups were immunostained for E-cadherin. As shown in Fig. 1e, the expression of E-cadherin was decreased in the lungs of BLM-treated mice and was recovered after administration of MLN. To examine the potential reparative ability of MLN on the damaged epithelium in BLM-treated mice, quantitative RT-PCR was performed to evaluate the expression levels of selected epithelial markers. We found that the mRNA levels of E-cadherin (Cdh1) and Sftpc were significantly decreased in BLM-treated mice and that these decreases were alleviated after treatment with MLN (Fig. 1f–g). Consistent with these results, MLN reversed the downregulation of E-cadherin at the protein level in BLM-treated mice (Fig. 1h). These data suggest that MLN alleviates lung injury and improves the survival of mice after treatment with BLM.

### MLN protects alveolar epithelial cells against apoptosis

**Fig. 2:**
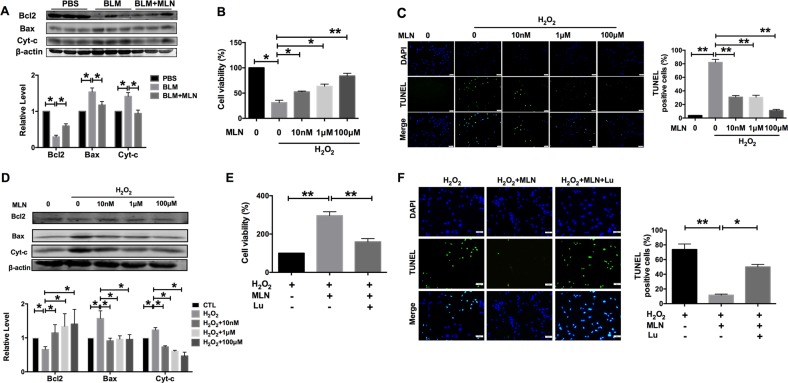
Melatonin attenuated apoptosis during lung injury. **a** Immunoblot analysis showed the expression of apoptosis-related proteins (Bcl2, Bax, and Cyt-c) in the PBS, BLM, and BLM + MLN groups. TC-1 cells were treated with H_2_O_2_ for 12 h with or without 10 nM, 1 μM, or 100 μM melatonin for 24 h. *P < 0.05. **b** An MTT assay was performed to assess TC-1 cells viability. *n* = 5; *P < 0.05, **P < 0.01. **c** Left, a TUNEL assay was performed to detect apoptotic cells, and TUNEL-positive cells were counted in TC-1 cells. Green, TUNEL-positive TC-1 cells; blue, DAPI. Scale bars, 50 μm. **P < 0.01. **d** Immunoblot analysis of the protein expression of Bcl2, Bax, and Cyt-c. *n* = 5; *P < 0.05. **e** Luzindole inhibited the effect of melatonin on cell viability in TC-1 cells, as indicated by an MTT assay. *n* = 6; **P < 0.01. **f** The number of TUNEL-positive cells was increased in the MLN + luzindole + H_2_O_2_ group compared with that in the MLN + H_2_O_2_ group. Green, TUNEL-positive TC-1 cells; blue, DAPI. Scale bars, 50 μm. Right, relative percentage of TUNEL-positive TC-1 cells. *P < 0.05, **P < 0.01

Apoptosis is one of the most common kinds of alveolar epithelial cell death during lung injury. The following study was designed to investigate whether MLN could protect alveolar epithelial cells against apoptosis. Western blot analyses showed that intratracheal instillation of BLM led to upregulated protein expression of the proapoptotic mediator Bax and downregulated expression of the antiapoptotic mediator Bcl2, accompanied by an increase in the mitochondrial release of Cyt-c, whereas those effects were nearly reversed after treatment with MLN (Fig. 2a). In vitro, TC-1 cells (alveolar epithelial cell line derived from mice) were treated with MLN (10 nM, 1 μM, and 100 μM) or PBS for 12 h before H_2_O_2_ (200 μM)-mediated impairment. MTT assays showed that H_2_O_2_ treatment significantly decreased cell viability and that this decrease was reversed by MLN in a concentration-dependent manner (Fig. 2b). Furthermore, MLN decreased the number of TUNEL-positive cells (Fig. 2c), accompanied by an increase in Bcl2 expression and a decrease in Bax and Cyt-c expression (Fig. 2d). To determine whether the beneficial effects of MLN on alveolar epithelial cells were mediated through the MLN receptor pathway, we used luzindole, a nonselective antagonist of the MLN receptor, to block MLN activity. We found that luzindole (10 μM) significantly inhibited the improvements in cell viability and apoptosis generated by MLN (100 μM) in H_2_O_2_-treated TC-1 cells (Fig. 2e, f). These results suggest that MLN suppresses the apoptosis of alveolar epithelial cells during lung injury.

### MLN inhibits alveolar epithelial cell senescence during lung injury

**Fig. 3:**
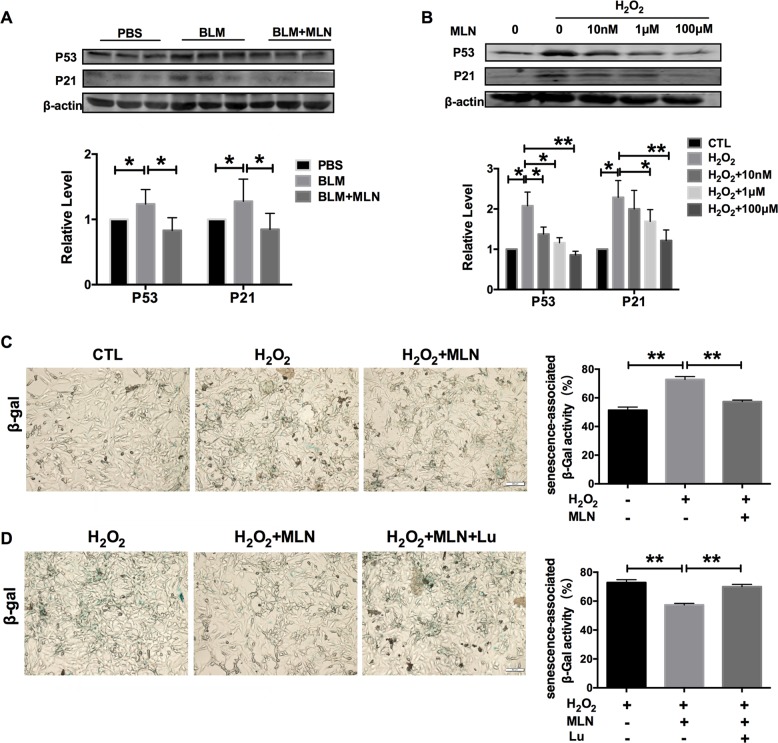
Melatonin inhibited senescence during lung injury. **a** Western blot analysis of the expression of p53 and p21 in whole lungs from mice in the PBS, BLM, and BLM + MLN groups. *P < 0.05. **b** Western blot analysis of p53 and p21 expression in TC-1 cells. *P < 0.05, **P < 0.01. **c** β-Gal staining showed increased senescence-associated β-gal activity in TC-1 cells 12 h after H_2_O_2_ treatment, and treatment with 100 μM melatonin reversed senescence in these cells. Scale bar, 50 μm. **P < 0.01. **d** Luzindole significantly blocked melatonin-induced senescence, as evidenced by staining for senescence-associated β-gal activity. Cells with green staining are senescent cells. Scale bar, 50 μm. **P < 0.01

Accumulating studies have shown that alveolar epithelial cell senescence contributes to the process of lung injury. We sought to verify whether MLN could protect against senescence in alveolar epithelial cells during BLM-induced lung injury. Immunoblotting was performed to detect the expression of senescence-associated proteins (p53 and p21) in vivo and in vitro, and β-gal staining was applied to examine the appearance of senescence in alveolar epithelial cells. We found that BLM resulted in dramatic lung senescence accompanied by the upregulation of p53 and p21 expression, which was abrogated after MLN administration (Fig. 3a). Furthermore, H_2_O_2_ treatment led to the upregulation of p53 and p21 expression, which was reversed by MLN (Fig. 3b). Interestingly, the beneficial effect of MLN on senescence was concentration-dependent. In addition, a significant increase in the number of β-gal-positive cells was observed in H_2_O_2_-treated TC-1 cells, and this increase was significantly reduced after treatment with 100 μM MLN (Fig. 3c). Moreover, luzindole treatment blocked the inhibitory effects of MLN on cellular senescence (Fig. 3d). Together, these data indicate that MLN can suppress alveolar epithelial cell senescence during lung injury.

### MLN improved mitochondrial function by inhibiting ROS production

**Fig. 4:**
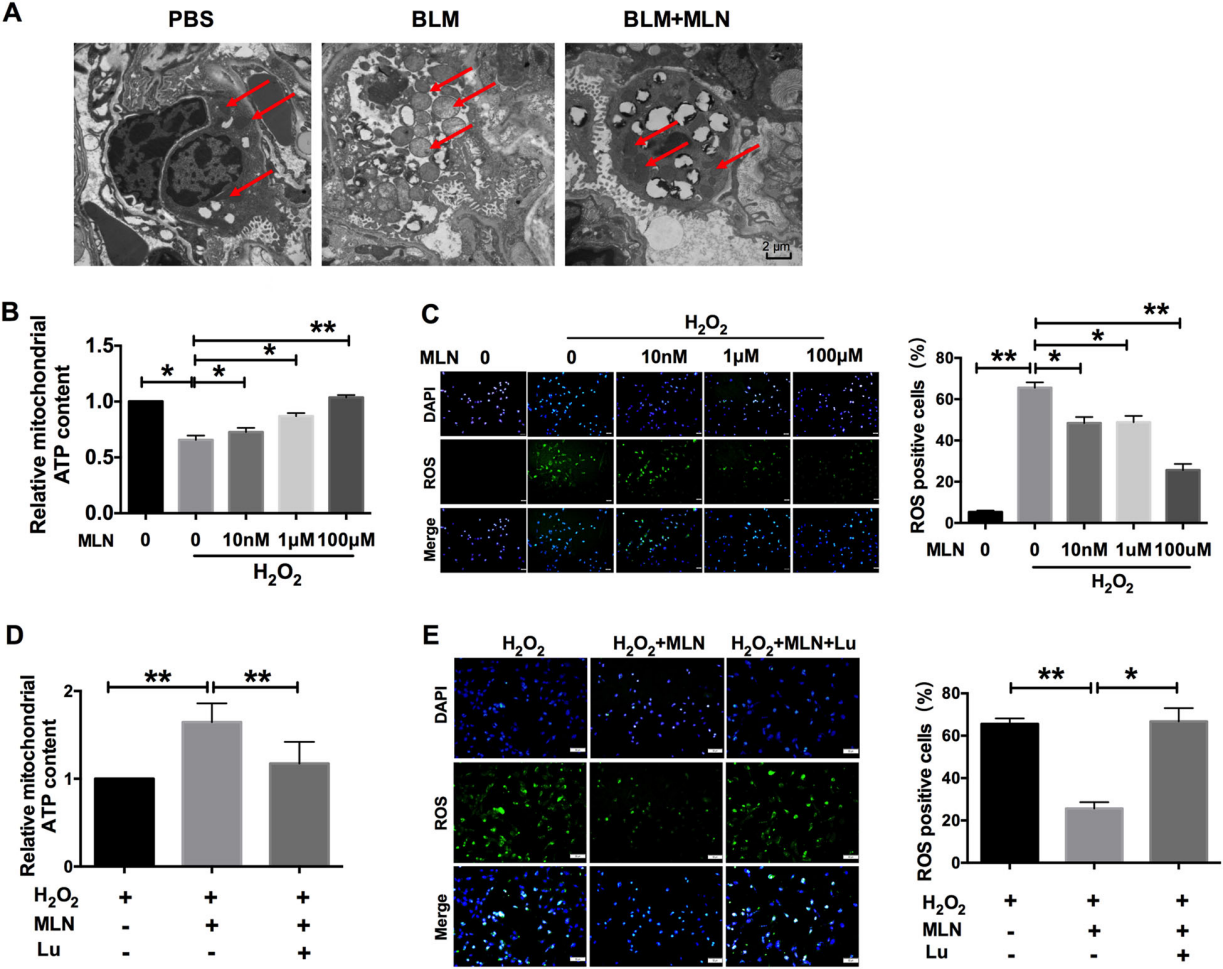
Melatonin reversed bleomycin-induced mitochondrial abnormalities in alveolar epithelial cells. **a** Representative transmission electron microscopy (TEM) images of lung sections from mice treated with PBS, BLM, or BLM + MLN. The red arrows indicate normal mitochondria or damaged and swollen mitochondria. **b** Cellular ATP content was measured in TC-1 cells incubated with the indicated concentrations of H_2_O_2_ and melatonin. Cells were lysed, and the ATP content was determined using an ATP assay and normalized to the protein concentration. *n* = 5; *P < 0.05, **P < 0.01. **c** Comparison of ROS production in TC-1 cells exposed to H_2_O_2_ with or without melatonin (10 nM, 1 μM, or 100 μM). Green, ROS; blue, DAPI. Scale bars, 50 μm. *P < 0.05, **P < 0.01. **d** Measurement of ATP content in TC-1 cells after exposure to H_2_O_2_, melatonin, and luzindole; the ATP content was normalized to the corresponding protein concentration. *n* = 5; **P < 0.01. **e** Effect of luzindole on ROS production in TC-1 cells. Green, ROS; blue, DAPI. Scale bars, 50 μm. *P < 0.05, **P < 0.01

It has been reported that apoptosis and senescence are mediated by mitochondria; thus, we investigated the ultrastructure of alveolar epithelial cells using transmission electron microscopy (TEM) at ×15,000 magnification. After BLM treatment, a large number of inflammatory cells were infiltrated into the alveolar cavity, mitochondrial edema was noticeable, and the endothelium of alveolar epithelial cells was not intact (Fig. 4a). However, MLN administration abolished the detrimental effects of BLM, maintaining the structure of alveolar epithelial cells and mitochondria (Fig. 4a). In a further evaluation of mitochondrial function, we found that H_2_O_2_ exposure significantly decreased the mitochondrial ATP content and that this decrease was mitigated by MLN in a concentration-dependent manner (Fig. 4b). These results suggest that MLN protects mitochondrial structure and function in alveolar epithelial cells during lung injury.

Oxidative stress has been reported to initiate and promote apoptosis and senescence by oxidizing mitochondrial membrane phospholipids and depolarizing the mitochondrial membrane potential^20^. To explore the underlying mechanism by which MLN improves mitochondrial function, we investigated ROS production and found that H_2_O_2_ exposure significantly enhanced ROS production, while treatment with MLN reduced ROS generation in a dose-dependent manner (Fig. 4c). As shown in Fig. 4d, e, luzindole treatment reversed the effect of MLN on the mitochondrial ATP content and ROS production. Taken together, these findings indicate that MLN improves mitochondrial function through inhibiting ROS production during lung injury.

### Apelin 13 benefits mitochondrial function

**Fig. 5:**
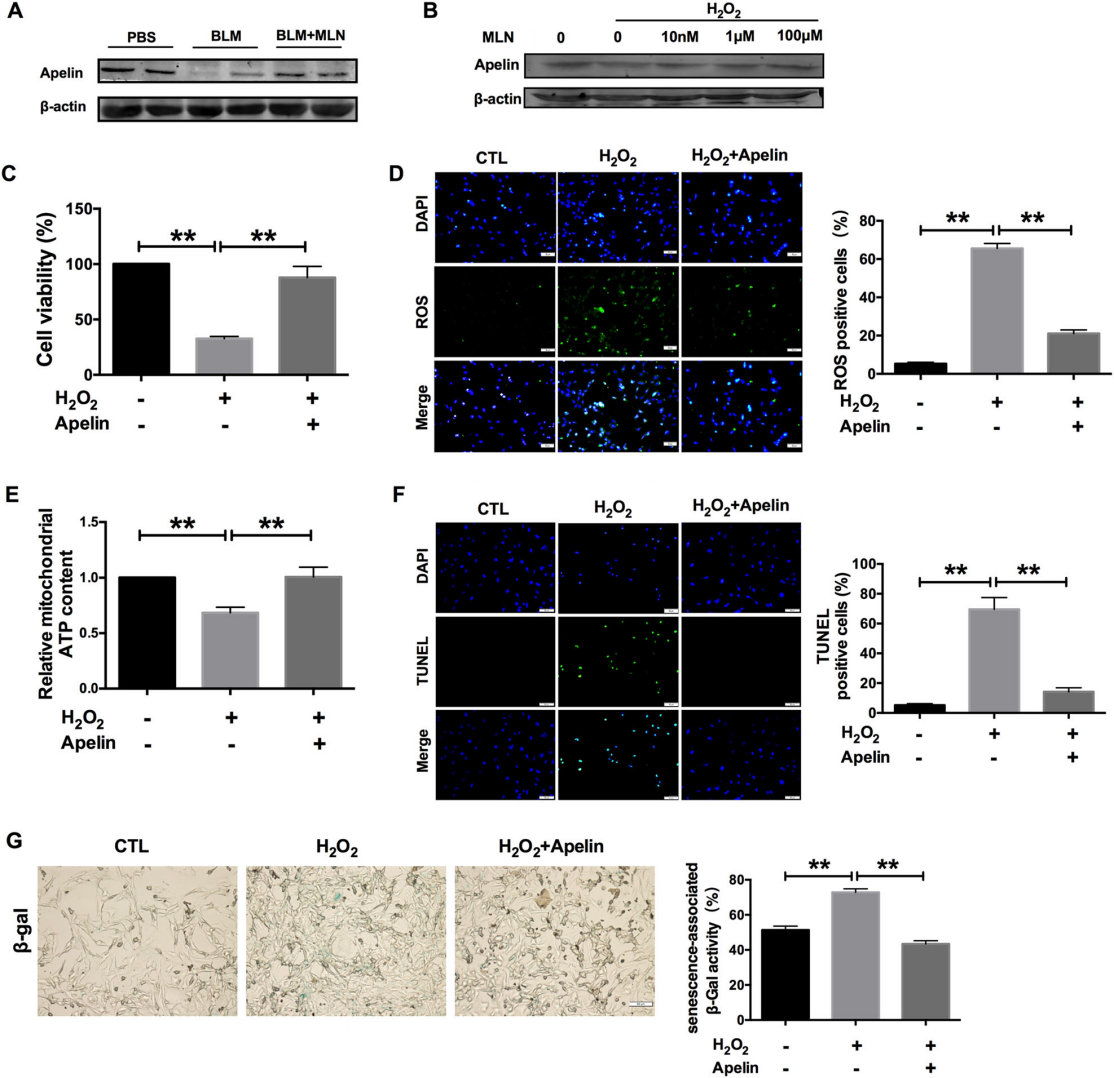
Apelin 13 participated in the process of by which melatonin prevents lung injury. **a** Western blot assays were performed to determine the protein levels of apelin 13 in lung tissues from mice in the PBS, BLM, and BLM + MLN groups. **b** The protein expression of apelin 13 in TC-1 cells after treatment with different concentrations of melatonin. **c** The MTT assay showed the effect of apelin 13 on the viability of H_2_O_2_-impaired TC-1 cells. *n* = 7; **P < 0.01. **d** Comparison of ROS production in TC-1 cells exposed to apelin 13. Green, ROS; blue, DAPI. Scale bars, 50 μm. **P < 0.01. **e** Apelin 13 impacted the mitochondrial ATP content in cells treated with H_2_O_2_. *n* = 5; **P < 0.01. **f** Representative images of the TUNEL assay in TC-1 cells are shown. Apelin 13 decreased the H_2_O_2_-induced increase in apoptosis. Green, TUNEL-positive TC-1 cells; blue, DAPI. Scale bars, 50 μm. **P < 0.01. **g** Apelin 13 significantly decreased cell senescence, as evidenced by staining for senescence-associated β-gal activity. Right, relative percentage of senescence-associated β-gal activity. **P < 0.01

Next, we explored the underlying mechanism by which MLN on mitochondrial function during lung injury. It has been reported that a structural analog of apelin, an endogenous ligand of the seven-transmembrane G protein-coupled receptor APJ, is involved in mitochondrial ROS generation and myocardial apoptosis^18^. We found that BLM significantly inhibited the expression of apelin and that this downregulation was reversed by MLN (Fig. 5a). Similarly, H_2_O_2_ dramatically inhibited apelin 13 expression, and treatment with 10 nM, 1 μM, and 100 μM MLN restored apelin expression (Fig. 5b). These data indicate that MLN promotes the expression of apelin 13 during lung injury.

To determine the role of apelin during lung injury, we treated TC-1 cells with 100 nM apelin for 24 h before treatment with H_2_O_2_. We found that treatment with apelin significantly alleviated H_2_O_2_-induced cell viability inhibition (Fig. 5c). Moreover, apelin dramatically inhibited H_2_O_2_-driven ROS production (Fig. 5d). As expected, apelin treatment increased mitochondrial ATP content and inhibited apoptosis and senescence in H2O2-treated TC-1 cells (Fig. 5e–g). These results suggest that apelin protects TC-1 cells against H_2_O_2_-induced injury and improves mitochondrial function.

### Apelin 13 is necessary for the protective effect of MLN on lung injury

**Fig. 6:**
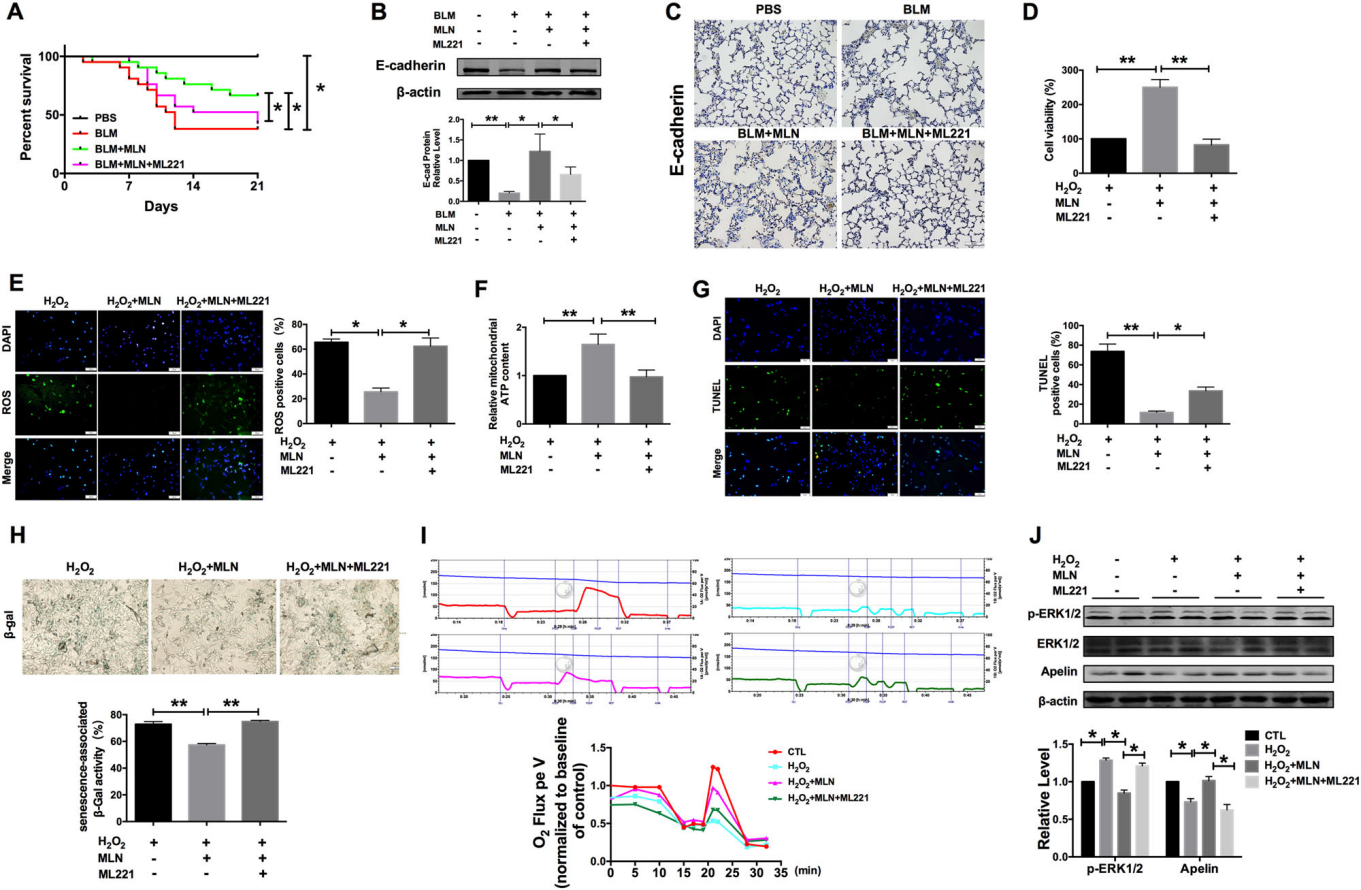
Blocking Apelin 13 by ML221 inhibited the effect of melatonin on alveolar epithelial cells. **a** Survival curves of mice after the injection of PBS, BLM, BLM + MLN, or BLM + MLN + ML221. *n* = 21; *P < 0.05. **b** E-cadherin, a marker of alveolar epithelial cells, was assessed by immunoblot analysis. *n* = 4; *P < 0.05, **P < 0.01. **c** The Immunohistochemical assay of E-cadherin in lung samples from mice in the PBS, BLM, BLM + MLN, and BLM + MLN + ML221 groups. Scale bar, 50 μm. **d** The effect of ML221 on cell viability was determined using an MTT assay. *n* = 7; **P < 0.01. **e** Comparison of ROS production in TC-1 cells exposed to ML221. Green, ROS; blue, DAPI. Scale bars, 50 μm. *P < 0.05. **f** ATP content in TC-1 cells treated with H_2_O_2_, melatonin, or ML221. The ATP content was normalized to the corresponding protein concentration. *n* = 5; **P < 0.01. **g** Treatment with ML221 inhibited the antiapoptotic effect of apelin 13. Green, TUNEL-positive TC-1 cells; blue, DAPI. Scale bars, 50 μm. *P < 0.05, **P < 0.01. **h** Left, TC-1 cells were treated with ML221 and were then fixed and stained for senescence-associated β-gal activity. **P < 0.01. **i** Representative profiles and summary data for oxygen consumption in TC-1 cells. The blue arrow indicates the oxygen concentration in the detection chamber. The blue line indicates the oxygen concentration in the detection chamber. **j** Western blot assays were performed to determine the protein levels of p-ERK1/2 and apelin 13 in TC-1 cells. *n* = 4; *P < 0.05

Then, we applied ML221, a functional antagonist of apelin 13, to further explore whether MLN protected against lung injury through modulating apelin 13 expression. The survival benefit conferred by MLN was blocked by injection of ML221 (Fig. 6a). As shown in Fig. 6b, c, ML221 inhibited MLN-induced upregulation of E-cadherin in BLM-treated mice. The MTT assay showed that ML221 significantly inhibited the MLN-induced increase in cell viability (Fig. 6d). In addition, ROS production was increased after ML221 treatment compared with that in the MLN-treated group (Fig. 6e). Moreover, ML221 treatment blocked the beneficial effects of MLN on mitochondrial ATP content, cell apoptosis, and senescence (Fig. 6f–h). We further measured mitochondrial respiratory function in TC-1 cells. H_2_O_2_ treatment significantly inhibited mitochondrial respiratory function, while ML221 inhibited the recovery of mitochondrial respiration mediated by MLN (Fig. 6i). Then, we explored the phosphorylation of ERK1/2 and found that MLN reversed the increase in the level of p-ERK1/2 induced by H_2_O_2_, while ML221 blocked the effect of MLN by inhibiting apelin 13 (Fig. 6j).

## Discussion

**Fig. 7:**
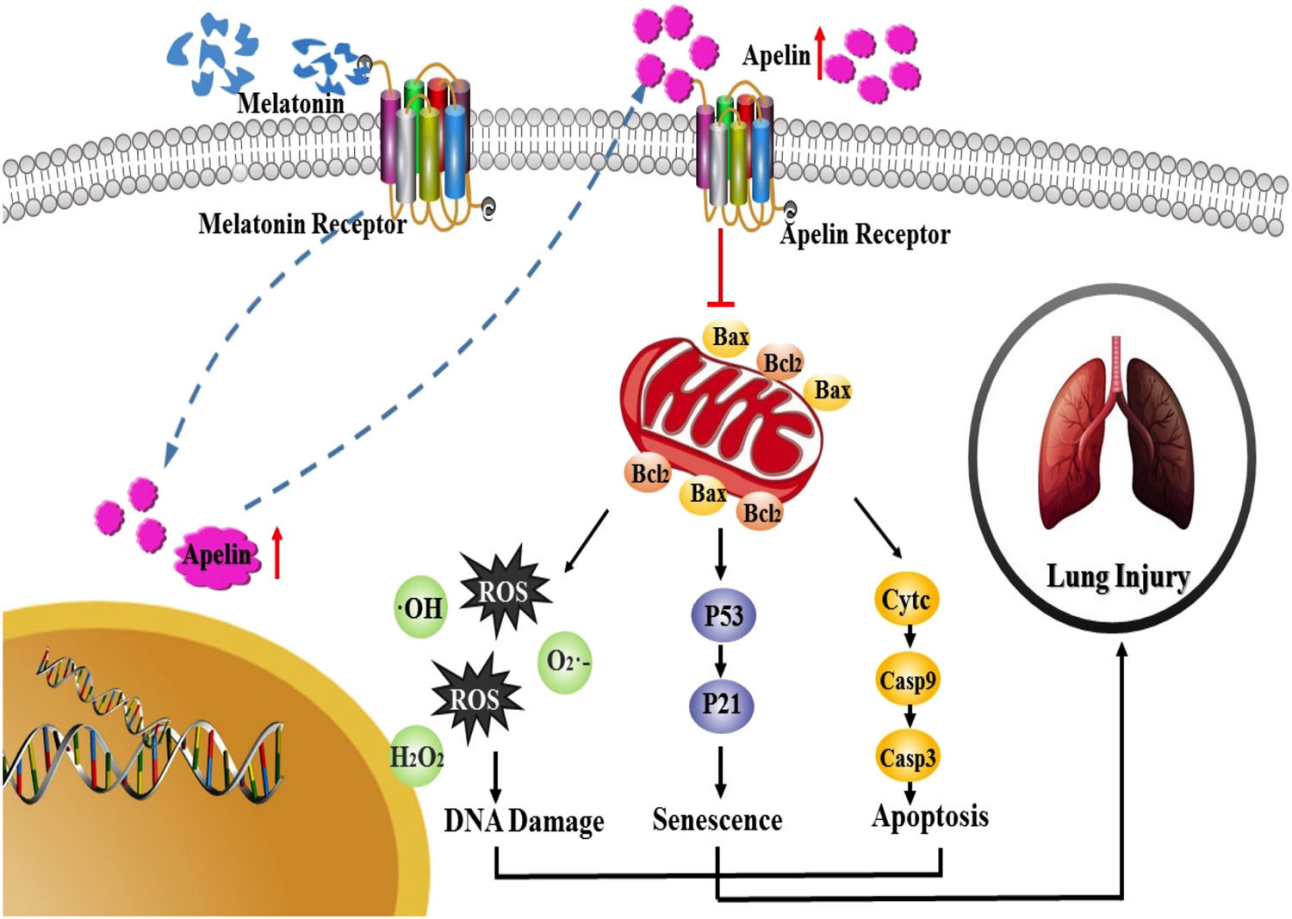
Schematic model describing the protective effect of melatonin on the restoration of mitochondrial homeostasis and function in AECIIs. Injury of AECIIs led to mitochondrial dysfunction, ROS release, and decreased ATP content. Melatonin supplementation modified injury through an epithelial protective effect via apelin 13 and its receptors, which resulted in the restoration of normal mitochondrial function and, eventually, rescued from mitochondria-regulated apoptosis and senescence

In the present study, we firstly clarified that the protective effect of MLN on lung injury is mediated via the prevention of ROS generation, apoptosis and senescence. In addition, the beneficial effect of MLN depends on mitochondrial function by regulating apelin 13. These findings revealed the molecular mechanism of MLN in pulmonary injury and suggested the potential clinical application of MLN in the treatment of pulmonary diseases (Fig. 7).

Lung injury occurs in many pulmonary diseases, such as acute respiratory distress syndrome and pulmonary fibrosis. Pulmonary edema and inflammatory cell infiltration are the characteristic histologic changes in lung injury, which is related to shock, acidosis, and ischemic injury^21^. AECIIs, the main functional cells that maintain pulmonary function, undergo apoptosis, and senescence during lung injury, which, in turn, exacerbates lung injury. However, no specific treatment for lung injury targeting lung alveolar epithelial cells is available.

Accumulating reports have shown that mitochondria not only supply chemical energy to power the cell but also modulate cellular calcium homeostasis, metabolism, and apoptosis. In addition, mitochondrial dysfunction has been recognized as a hallmark of epithelial cell injury in pulmonary fibrosis^22^. Thus, maintaining the structural and functional integrity of mitochondria is vital for the homeostasis of lung alveolar epithelial cells and even for lung function. A recent study showed that thyroid hormone, which played a protective role against lung injury, could protect alveolar epithelial cells and restore mitochondrial function^23^. In addition, MLN has been found to promote mitophagy and improve mitochondrial homeostasis^24^. MLN pretreatment enhances the therapeutic effects of exogenous mitochondria against hepatic ischemia-reperfusion injury in rats through the suppression of mitochondrial permeability transition^25^. Thus, we proposed a hypothesis that MLN protects against lung injury by improving mitochondrial function. We found that MLN administration attenuated BLM-induced death in mice, inhibited inflammation, and repaired epithelial function. Furthermore, MLN treatment increased the number of mitochondria in alveolar epithelial cells. These data suggest that MLN protects against BLM-induced lung injury and promotes mitochondrial function.

Apoptosis and senescence are two important outcomes of alveolar epithelial cells during lung injury, and both of these processes are associated with mitochondrial function^26^. When mitochondrial dysfunction occurs, ROS are produced at a high level, which causes cytochrome C efflux and induces apoptosis^27^. Cells with mitochondrial dysfunction had lower NAD+/NADH ratios, which caused growth arrest and prevented IL-1-associated SASP through AMPK-mediated p53 activation^28^. In our study, we found that H_2_O_2_ treatment resulted in mitochondrial dysfunction in alveolar epithelial cells, with decreased ATP production and increased ROS production, which were reversed byMLN in a dose-dependent manner. However, blocking the binding betweenMLN and its receptor using luzindole abolished the protective effect of MLN.

Further studies explored the mechanism by which MLN improves mitochondrial function. Accumulating reports showed that apelin 13 acts as a key protective modulator of lung diseases^29,30^ and that apelin 13 promotes mitochondrial biogenesis^31^ and increases mitochondrial oxidative capacity^32^. Here, we found that MLN increased the expression of apelin 13. Consistent with the results of previous studies, apelin 13 treatment increased alveolar epithelial cell viability, elevated ATP production, decreased ROS levels, suppressed apoptosis, and inhibited senescence. Moreover, when apelin 13 activation was blocked using ML221, MLN no longer protected mitochondrial function, resulting in significant ROS production, apoptosis, and senescence.

Our study is limited by the following factors. First, the role of apelin 13 in the protective effects of MLN on lung injury was not further verified in vivo, and the relationship between MLN and apelin 13 remains to be further explored. Second, we did not determine endogenous MLN concentrations or circadian phase in any of the experimental subjects. Although we performed the intraperitoneal injections of MLN at noon, when endogenous MLN secretion is the lowest, there is a lack of effective evidence for the optimal time and dose of MLN as a clinical medication. Circadian rhythm tracking would be an important addition to future studies by informing the treatment approach to achieve maximal benefit. More work is needed to explore MLN as a treatment for lung injury.

In conclusion, MLN increased the expression of apelin 13 and inhibited the production of ROS, which recovered mitochondrial function, leading to a reduction in apoptosis, and senescence during lung injury (Fig. 7). Taken together, our results strongly suggest that approaches using MLN should be further studied in lung diseases.
